# Success in Business

**Published:** 1872-04

**Authors:** 


### SUCCESS IN BUSINESS

Prevents an incalculable amount of suffering, sickness, and premature death, and yet many young men fail of that success, from not starting out in life with an unalterable determination to adhere conscientiously and tenaciously to a few sterling, general principles of conduct.

1st. Never misrepresent.

2d. Let your spoken word be more reliable than your written bond.

3d. Never pay a bill without taking a receipt in full.

4th. Treat a customer as your friend, and never allow him to be disappointed.

5th. Keep down expenses.

6th. Invest profits safely.

7th. Live within your income.

8th. If hard run for money, let your wife know it. There were invitations out for a splendid party on the very night Professor Webster killed his friend and burnt him up, for the sake of money!

9th. Don't boast of your business. Said a man one day to the elder Astor, "Why is it that you have made so much money and I none, although I have been as temperate, as industrious, and as economical as you?"

"You talk too much," replied the millionaire.

A man came in to-day, who, a few years ago, was in the receipt of twenty-five hundred dollars a year; and now, at the age of sixty years, so much of an invalid that he could not walk a hundred yards without resting, to beg half a dollar to get to-morrow's bread for himself, and wife, and three helpless grandchildren.

We knew him in his prosperous days. He spent all he made, and invested nothing; as if he could never leave his place; as if he could always be well and young enough to perform the duties of his position.

